# Supplementary material for: DOT1L-mediated murine neuronal differentiation associates with H3K79me2 accumulation and preserves SOX2-enhancer accessibility
Source: Nat Commun. 2020 Oct 15;11:5200. doi: 10.1038/s41467-020-19001-7 (PMC7562744; doi:10.1038/s41467-020-19001-7)
Supplement: Supplementary file 1 — Supplementary Information [file 41467_2020_19001_MOESM1_ESM.pdf]

DOT1L-mediated Murine Neuronal Differentiation Associates with H3K79me2 Accumulation and Preserves SOX2-Enhancer Accessibility

Ferrari et al.

Supplementary Figure 1

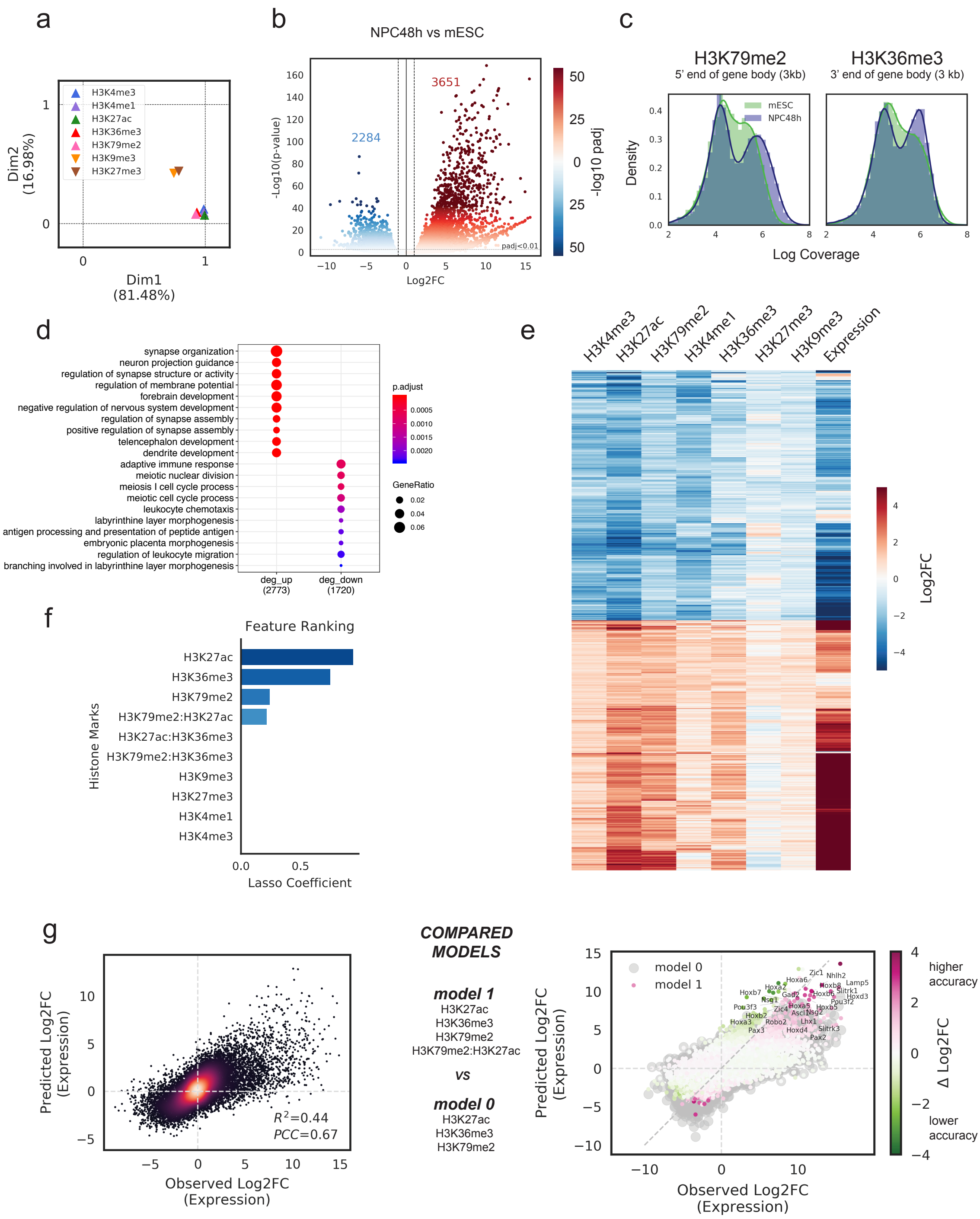

Neuronal differentiation correlates with relative transcriptional and epigenetic activation

a) Graph showing the contribution of each group of features (most variable 2kb bins for each histone modification) to the first and second dimension of the multiple factor analysis in Figure 1b, lower panel.

b) Volcano plot summarizing differential expression analysis for the contrast NPC48h vs mESC. Genes are color-coded according to the -log10(adjusted p-value), genes with decreased expression in blue and with increased expression in red. The dotted lines denote the thresholds used in this study (abs(log2 fold-change)>1, adjusted p-value<0.01).

c) Distribution of H3K79me2 and H3K36me3 normalized coverage (relative log expression (RLE) normalization on background regions), computed over 3kb window downstream of TSS of protein coding genes and 3kb upstream of TTS of protein coding genes respectively, in mESC (green) and NPC48h (blue). TSS: transcription start site, TTS: transcription termination site.

d) Top 10 most significant over-represented GO terms (adjusted p-value < 0.05) based on significantly upregulated (left) and downregulated (right) genes (abs(log2 fold-change) > 1, adjusted p-value < 0.01) in the comparison between ES-derived NPC48h and mESC.

e) Heatmap showing the log2 fold-change (NPC48h vs mESC) of histone modifications (H3K4me3, H3K27ac, H3K4me1: narrow promoter (TSS ±1kb); H3K79me2, H3K27me3, H3K9me3: extended promoter (TSS -1kb,+3kb); H3K36me3: transcription termination site (TTS - 3kb,+0.5kb)) and the log2 fold-change in expression (NPC48h vs mESC) of the corresponding gene. We show the top 1000 genes with increased and decreased expression.

f) Lasso regression coefficients are used to rank all input features. We retain a sparse model to predict transcriptional changes with three histone marks (H3K27ac, H3K36me3, H3K79me2) and an interaction term H3K79me2:H3K27ac.

g) Fit of the multiple linear regression model. Observed vs predicted log2 fold-changes of gene expression (NPC48h vs mESC) as predicted through the linear combination of log2 fold-changes of H3K27ac, H3K36me3, H3K79me2 and H3K27ac:H3K79me2 (R^2=0.44) (left panel). Predictions for different genes are differently affected by the interaction term (right panel). The biggest improvement in predictive accuracy is achieved for genes that are known targets of retinoic acid mediated neuronal differentiation (Hoxa, Hoxb cluster genes, Ascl1, Zic1, Zic4, Pou3f2, Pou3f3, Nhlh2, Lhx1).

Supplementary Figure 2

a

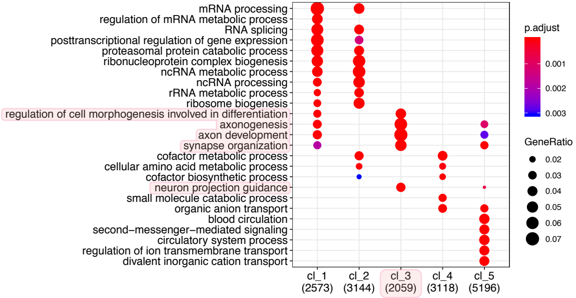

Gain of H3K79me2 enrichment during neuronal differentiation marks neuronal genes

a) Gene ontology term enrichment analysis for each of the five clusters of genes of Figure 2d. The size of each dot is proportional to the gene ratio for each significant GO term, while the color maps the adjusted p-value for the over-representation test.

Supplementary Figure 3

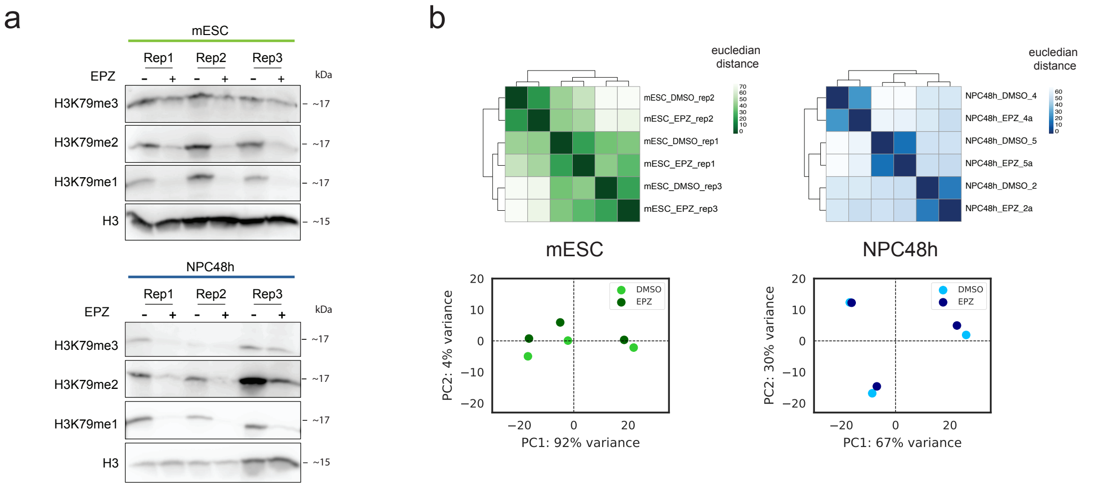

EPZ treatment depletes H3K79me2 with minor transcriptional changes

a) EPZ5676 treatment for 48h reduces the level of H3K79me1, -me2 and -me3 in mESC and NPC48h. Immunoblotting of H3K79me1/me2/me3 and H3 (loading control) of EPZ-treated and DMSO-treated mESC (upper panel, green) and NPC48h (lower panel, blue).

b) Top panel: hierarchical clustering of mESC (green, left) and NPC48h (blue, right) samples based on the euclidean distance of the rlog transformed transcriptome. Darker shades are used for closer samples. Lower panel: principal component analysis of mESC (left, green) and NPC48h (right, blue) samples based on the top 500 most variable genes (rlog transformed counts).

Supplementary Figure 4

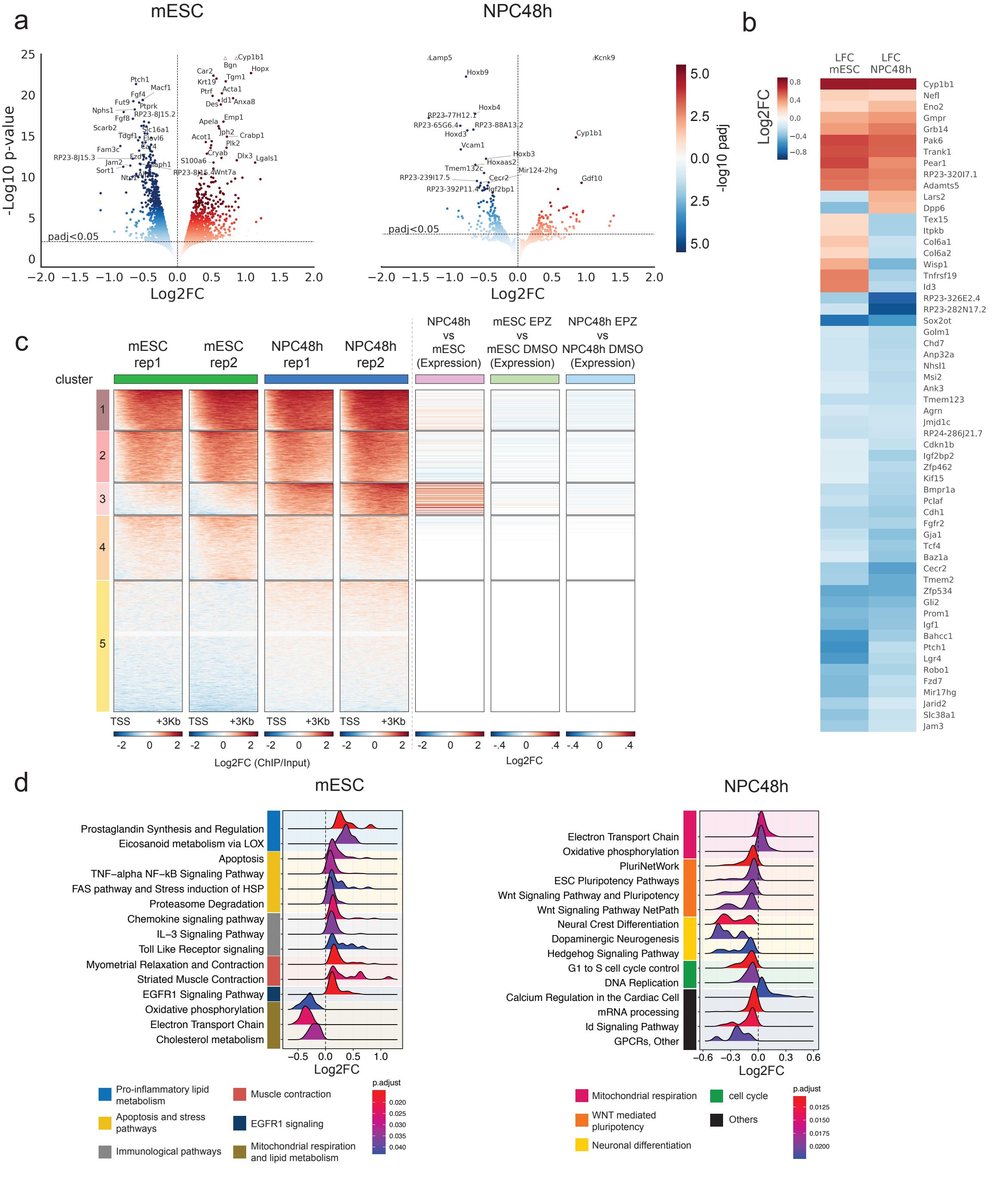

DOT1L inhibition induces cell-type specific transcriptional changes

a) EPZ treatment alters the transcriptome in mESC and NPC48h. Volcano plots showing log2 fold-change (x-axis) and -log10(p-value) (y-axis) of all genes tested for differential expression for the contrast EPZ vs DMSO treated mESC (left panel) and NPC48h (right panel). Genes are color-coded according to the respective adjusted p-value. Shade of blue is used for genes with decreased expression, while shade of red is used for genes with increased expression. The topmost significant gene names are shown.

b) Heatmap showing the log2 fold-change in expression of the 58 common differentially expressed genes in mESC and NPC48h upon DOT1L inhibition.

c) Heatmap showing the five clusters of protein coding genes identified in Figure 2d, based on the signal of H3K79me2 in mESC and NPC48h 3kb downstream of TSS (first four columns). Next, the expression log2 fold change for the contrasts NPC48h vs mESC (fifth column), mESC EPZ-treated vs mESC DMSO-treated (sixth column), and NPC48h EPZ-treated vs NPC48h DMSO-treated (seventh column). No strong association is observed between gain of H3K79me2 during differentiation and transcriptional deregulation as a consequence of EPZ treatment (φ coefficient = 0.046, 65 DEG of 2170 genes gaining H3K79me2 during neuronal differentiation).

d) Transcriptional deregulation induced by EPZ treatment affects groups of genes that are functionally coherent. Ridgeplot representing the expression log2 fold-change (EPZ vs DMSO) distribution (x-axis) of the leading-edge genes from the top 15 most significant pathways (ranked on adjusted p-value), identified by running GSEA against the wikiPathways database, in mESC (left panel) and NPC48h (right panel). Each distribution is shaded according to adjusted p-value of the associated pathway. Pathways are grouped and color-coded according to functional similarity.

Supplementary Figure 5

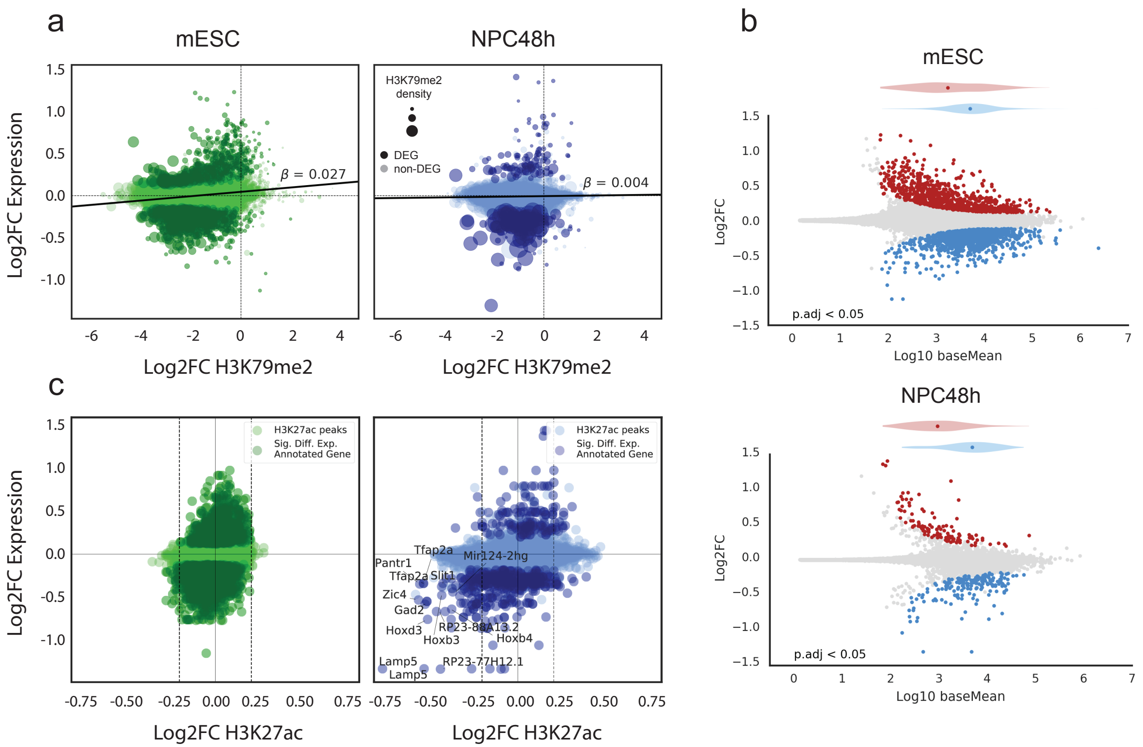

Inhibition of DOT1L does not affect the overall epigenome and transcriptome

a) Simple linear regression of differential expression induced by EPZ treatment compared to control on differential H3K79me2 (estimation based on RELACS data on a 3kb window downstream of TSS), for mESC (left panel, green) and NPC48h (right panel, blue). The size of each dot is proportional to the H3K79me2 density. A darker shade is used for differentially expressed genes (DEG) upon DOT1L inactivation.

b) MA plot showing log10 of the mean count (x-axis) vs expression log2 fold-change (y-axis) as computed by DESeq2, for each gene for the contrast EPZ vs DMSO treated cells. Genes significantly increasing and decreasing in expression (adjusted p-value < 0.05) are shown in red and blue respectively. Horizontal violin plots show the Log10 mean count distribution of significantly upregulated and downregulated genes. Log2 fold-change are shrunken using apeglm. MA plot of mESC is shown on the left, while MA plot of NPC48h is shown on the right.

c) H3K27ac peaks are depleted in a targeted set of genes in NPC48h upon DOT1L inhibition. Scatterplot showing the association between log2 fold-change of H3K27ac peaks (x-axis) and the expression log2 fold-change of annotated genes (y-axis) upon EPZ treatment for mESC (left, green) and NPC48h (right, blue). A peak is annotated to a gene if the peak overlaps any feature of the gene (promoter-TSS, introns, exons, TTS) or if it is proximal to the TSS/TTS (± 1kb). Each dot represents a H3K27ac peak. Darker dots represent H3K27ac peaks overlapping differentially expressed genes (adjusted p-value < 0.05) upon DOT1L inhibition. Peaks showing a significant loss of H3K27ac in NPC48h upon EPZ treatment are annotated with the gene symbol of the corresponding overlapping gene.

Supplementary Figure 6

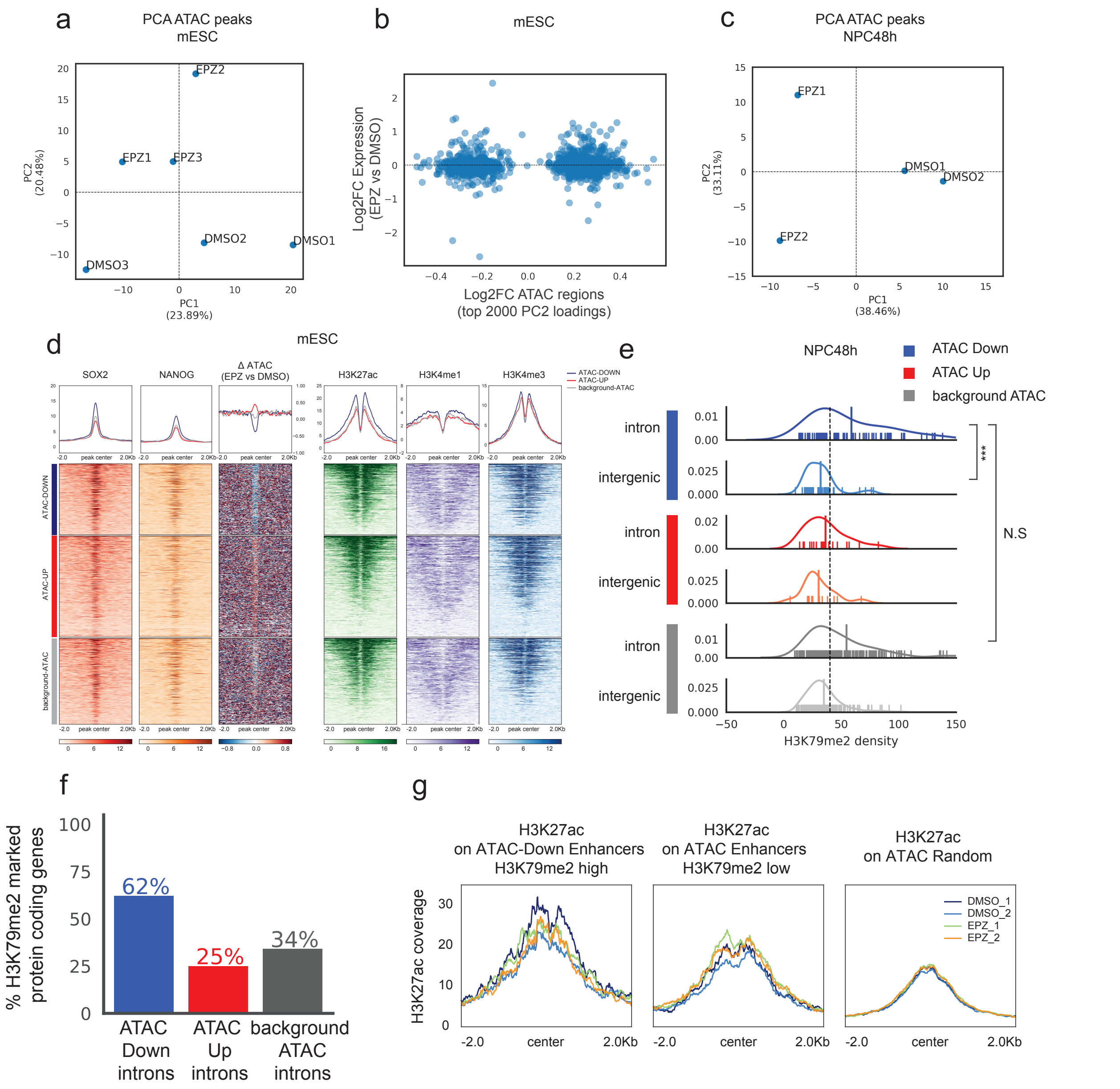

Presence of H3K79me2 is not predictive for decreased accessibility upon EPZ treatment and altered H3K79me2 do not correlate with altered H3K27ac on enhancer regions

a) Principal component analysis of rlog transformed ATAC peaks coverage in mESC.

b) Scatterplot showing accessibility change of the top 2000 ATAC peaks with higher loading absolute value on PC2 for mESC PCA (x-axis) and the expression log2 fold-change of annotated genes proximal/overlapping each dynamic ATAC peak (y-axis) upon DOT1L inhibition. No association is found between dynamic ATAC and expression change in mESC.

c) Principal component analysis of rlog transformed ATAC peaks coverage in NPC48h.

d) Metaprofile and heatmap of SOX2 and NANOG profile in mESC 36,37 over open regions losing accessibility (ATAC-Down), gaining accessibility (ATAC-Up) and unaffected (background-ATAC) upon EPZ treatment in mESC. Metaprofile and heatmap of the corresponding log2 ratio of EPZ vs DMSO ATAC-seq signal, H3K27ac, H3K4me1 and H3K4me3 input subtracted coverage of DMSO treated sample.

e) Rug plot showing the distribution of H3K79me2 density on intergenic and intronic ATAC peaks with decreased accessibility upon EPZ treatment (ATAC-Down, blue), with increased accessibility upon EPZ treatment (ATAC-Up, red), and with no effect by EPZ treatment (background-ATAC, grey). The mean of each group is represented by the highest spike. While H3K79me2 density is significantly higher in intronic compared to intergenic regions showing decreased accessibility (p-value = 0.113), no difference is observed between intronic regions losing accessibility and 1000 randomly chosen accessible intronic regions (p-value = 4.999 x 10-7). To test for significant differences in the mean, we used a two-sided non-parametric Mann-Whitney U-test. N.S: not significant; *** : p-value < 0.001.

f) Barplot showing the percentage of H3K79me2 positive protein coding genes over the total number of protein coding genes annotated with at least one intronic ATAC peak showing decreased accessibility (ATAC Down introns), increased accessibility (ATAC Up introns) and non-dynamic accessibility (background-ATAC introns) upon EPZ treatment with respect to DMSO control in NPC48h.

g) Metaprofiles of H3K27ac over enhancers regions, stratified on H3K79me2 density. From left to right, we show H3K27ac profile on ATAC Down regions with high H3K79me2 (H3K79me2 density > 45), on ATAC enhancers with low H3K79me2 (H3K79me2 density < 45), and on random ATAC regions as background. Regions are aligned on the ATAC peak center.

SUPPLEMENTARY TABLES

Supplementary Table 1: RELACS custom barcodes

Sample	RELACS barcode
mESC_DMSO_rep1	TTCGCTCT
mESC_DMSO_rep2	ACGTGTAC
mESC_EPZ_rep1	TACCGATG
mESC_EPZ_rep2	TTGGTTGG
NPC48h_DMSO_rep1	CCTCTCAA
NPC48h_DMSO_rep2	TTGTGGCT
NPC48h_EPZ_rep1	CCGAATAC
NPC48h_EPZ_rep2	TGTGATCG

Supplementary Table 2: Antibody details

Histone modification	Ab details (company, product ID, lot)	Concentration ($\mu g\ \mu L^{-1}$)	Amount per ChIP
H3K27ac	Diagenode, C1541096, lot A1723-041D	2.84	1μg / 100,000 cells
H3K27me3	Diagenode, C15410195, lot A1811-001P	1.91	1μg / 100,000 cells
H3K36me3	Diagenode, C15410192, lot A1847-001P	1.19	1μg / 100,000 cells
H3K4me1	Diagenode, C15410194, lot A1863-001D	0.91	1μg / 100,000 cells
H3K4me3	Diagenode, C15410003, lot A5051-001P	1	1μg / 100,000 cells
H3K79me2	Abcam, ab3594	2	1μg / 100,000 cells
H3K9me3	Diagenode, C15410193, lot A1671-001P	2.69	1μg / 100,000 cells

Supplementary Table 3: Antibody details for immunoblotting

**Histone modification**	**Ab details (company, product ID)**	**Concentration ($\mu g\ \mu L^{-1}$)**	**Dilution**
H3	Abcam, ab12079	0.9	1:1000
H3K79me1	Abcam, ab177183	2.4	1:20000
H3K79me2	Abcam, ab3594	1	1:1000
H3K79me3	Abcam, ab2621	0.7	1:1000
