# Supplementary material for: DOT1L-mediated murine neuronal differentiation associates with H3K79me2 accumulation and preserves SOX2-enhancer accessibility
Source: Nat Commun. 2020 Oct 15;11:5200. doi: 10.1038/s41467-020-19001-7 (PMC7562744; doi:10.1038/s41467-020-19001-7)
Supplement: Supplementary file 3 — Reporting Summary [file 41467_2020_19001_MOESM3_ESM.pdf]

nature research

Corresponding author(s): Thomas Manke, Tanja Vogel

Last updated by author(s): Aug 5, 2020

Reporting Summary

Nature Research wishes to improve the reproducibility of the work that we publish. This form provides structure for consistency and transparency in reporting. For further information on Nature Research policies, see Authors & Referees and the Editorial Policy Checklist .

Statistics

For all statistical analyses, confirm that the following items are present in the figure legend, table legend, main text, or Methods section.

n/a	Confirmed	
☐	☒	The exact sample size (*n*) for each experimental group/condition, given as a discrete number and unit of measurement
☐	☒	A statement on whether measurements were taken from distinct samples or whether the same sample was measured repeatedly
☐	☒	The statistical test(s) used AND whether they are one- or two-sided<br>*Only common tests should be described solely by name; describe more complex techniques in the Methods section.*
☐	☒	A description of all covariates tested
☐	☒	A description of any assumptions or corrections, such as tests of normality and adjustment for multiple comparisons
☐	☒	A full description of the statistical parameters including central tendency (e.g. means) or other basic estimates (e.g. regression coefficient) AND variation (e.g. standard deviation) or associated estimates of uncertainty (e.g. confidence intervals)
☐	☒	For null hypothesis testing, the test statistic (e.g. *F*, *t*, *r*) with confidence intervals, effect sizes, degrees of freedom and *P* value noted<br>*Give P values as exact values whenever suitable.*
☐	☒	For Bayesian analysis, information on the choice of priors and Markov chain Monte Carlo settings
☐	☒	For hierarchical and complex designs, identification of the appropriate level for tests and full reporting of outcomes
☐	☒	Estimates of effect sizes (e.g. Cohen's *d*, Pearson's *r*), indicating how they were calculated

Our web collection on statistics for biologists contains articles on many of the points above.

Software and code

Policy information about availability of computer code

Data collection	A complete list of open source code used in this study is available at https://github.com/FrancescoFerrari88/code_DOT1L_paper/tree/master/conda_environments. Here is a list of the main tools used: snakePipes (v. 1.1.1), MACS2 (v. 2.2.6), DESeq2 (v. 1.22.1), featureCounts (v. 1.6.4), Homer (v. 4.10), UROPA (v. 3.1.0), GimmeMotifs (v. 0.13.1), deeptools (v. 3.1.2), deepStats (v. 0.3.1), Ultraheatmap (v. 1.3.0), FactoMineR (v. 1.41), scikit-learn (v. 0.19.1), statsmodels (v. 0.9.0), clusterProfiler (v. 3.10.1), ChromHMM (v. 1.15), pymc3 (v. 3.6), Python (v. 3.6), R (v. 3.5), ImageJ (v. 1.52k).
Data analysis	A complete collection of custom code used to analyze the data is available at https://github.com/FrancescoFerrari88/code_DOT1L_paper. The code is structured according to the images it generates.

For manuscripts utilizing custom algorithms or software that are central to the research but not yet described in published literature, software must be made available to editors/reviewers. We strongly encourage code deposition in a community repository (e.g. GitHub). See the Nature Research guidelines for submitting code & software for further information.

Data

Policy information about availability of data

All manuscripts must include a data availability statement. This statement should provide the following information, where applicable:
- Accession codes, unique identifiers, or web links for publicly available datasets
- A list of figures that have associated raw data
- A description of any restrictions on data availability

SOX2 binding profiles in brain-derived NPC were retrieved from the following public GEO repository: GSE90559 [https://www.ncbi.nlm.nih.gov/geo/query/acc.cgi?acc=GSE90559]. SOX2 and NANOG binding in mESC were retrieved from public GEO entries GSM1050291 [https://www.ncbi.nlm.nih.gov/geo/query/acc.cgi?acc=GSM1050291] and GSM1847493 [https://www.ncbi.nlm.nih.gov/geo/query/acc.cgi?acc=GSM1847493] respectively. Raw data and normalized bigWig tracks generated in this work were deposited to GEO and are available for download using the following accession number: GSE135318 [https://www.ncbi.nlm.nih.gov/geo/query/acc.cgi?acc=GSE135318].

Field-specific reporting

Please select the one below that is the best fit for your research. If you are not sure, read the appropriate sections before making your selection.

- [x] Life sciences
- [ ] Behavioural & social sciences
- [ ] Ecological, evolutionary & environmental sciences

For a reference copy of the document with all sections, see nature.com/documents/nr-reporting-summary-flat.pdf

Life sciences study design

All studies must disclose on these points even when the disclosure is negative.

Sample size

Sample sizes were determined based on the recommendations for high-throughput sequencing experiments (minimal 2-3 replicates per condition). Given the high number of epigenetic marks included in this study, we generated 2 replicated per cell-type.

Data exclusions

No data was excluded.

Replication

All sequencing experiments presented in this paper have been conducted once, with the appropriate biological replicates included in the experimental run. Immunoblotting was replicated at least twice, with each replicate including all biological replicates shown in the study. Sox2 ChIP followed by qPCR was performed once.

Randomization

Randomization was not relevant to the present study because we worked with cultured cells grown in a highly controlled and homogeneous environment.

Blinding

Authors contributing to the experimental validation involving SOX2-ChIP followed by qPCR, were blind with respect to the NGS predictions. For example, the researcher conducting the experimental validation was unaware of which loci were negative controls and which were differentially accessible.

Reporting for specific materials, systems and methods

We require information from authors about some types of materials, experimental systems and methods used in many studies. Here, indicate whether each material, system or method listed is relevant to your study. If you are not sure if a list item applies to your research, read the appropriate section before selecting a response.

Materials & experimental systems

n/a	Involved in the study	
[ ]	[x]	Antibodies
[ ]	[x]	Eukaryotic cell lines
[x]	[ ]	Palaeontology
[x]	[ ]	Animals and other organisms
[x]	[ ]	Human research participants
[x]	[ ]	Clinical data

Methods

n/a	Involved in the study	
[ ]	[x]	ChIP-seq
[x]	[ ]	Flow cytometry
[x]	[ ]	MRI-based neuroimaging

Antibodies

Antibodies used

RELACS ChIP-SEQ:
H3K27ac: Diagenode, C1541096, lot A1723-041D, concentration: 2.84 µg/µL, used: 1ul per 100,000 cells
H3K27me3: Diagenode, C15410195, lot A1811-001P, concentration: 1.91 µg/µL, used: 1ul per 100,000 cells
H3K36me3: Diagenode, C15410192, lot A1847-001P, concentration: 1.19 µg/µL, used: 1ul per 100,000 cells
H3K4me1: Diagenode, C15410194, lot A1863-001D, concentration: 0.91 µg/µL, used: 1ul per 100,000 cells
H3K4me3: Diagenode, C15410003, lot A5051-001P, concentration: 1 µg/µL, used: 1ul per 100,000 cells
H3K79me2: Abcam, ab3594, concentration: 2 µg/µL, used: 1ul per 100,000 cells
H3K9me3: Diagenode, C15410193, lot A1671-001P, concentration: 2.69 µg/µL, used: 1ul per 100,000 cells

WB:
H3: Abcam, ab12079, concentration: 0.9 µg/µL, dilution: 1:1000
H3K79me1, Abcam, ab177183, concentration: 2.4 µg/µL, dilution: 1:20000
H3K79me2, Abcam, ab3594, concentration: 1 µg/µL, dilution: 1:1000
H3K79me3, Abcam, ab2621, concentration: 0.7 µg/µL, dilution: 1:1000

SOX2ChIP-qPCR:

	SOX2: anti-SOX2, Santa Cruz, sc-365964, 2µg per 500 µg of total protein extracts;<br>mouse IgG, Diagenode, C15400001-15, dilution:1:500
Validation	All antibodies used in this study were previously validated as part of the German Epigenome Project (http://www.deutsches-epigenom-programm.de/), via ChIP-seq and immunoblotting.

Eukaryotic cell lines

Policy information about cell lines

Cell line source(s)	Dot1l-HA-FLAG C57BL/6 mouse embryonic stem cells and in-vitro derived neural progenitor cells (according to Bibel at al. 2008). Dot1l-HA-FLAG were generated by inGenious Targeting Laboratory, and purchased from the same vendor.
Authentication	Cells were authenticated via immunostainings, RNA-seq and ChIP-seq
Mycoplasma contamination	The cells were not tested for mycoplasma contamination
Commonly misidentified lines<br>(See ICLAC register)	No commonly misidentified cell lines were used in the study.

ChIP-seq

Data deposition

- [x] Confirm that both raw and final processed data have been deposited in a public database such as GEO.
- [ ] Confirm that you have deposited or provided access to graph files (e.g. BED files) for the called peaks.

Data access links<br>*May remain private before publication.*	GSE135318 (publicly accessible as of 1 August 2020)
Files in database submission	mESC_DMSO_rep1_H3K27ac_merged_R1.fastq.gz<br>mESC_DMSO_rep2_H3K27ac_merged_R1.fastq.gz<br>mESC_DMSO_rep1_H3K27me3_merged_R1.fastq.gz<br>mESC_DMSO_rep2_H3K27me3_merged_R1.fastq.gz<br>mESC_DMSO_rep1_H3K36me3_merged_R1.fastq.gz<br>mESC_DMSO_rep2_H3K36me3_merged_R1.fastq.gz<br>mESC_DMSO_rep1_H3K4me1_merged_R1.fastq.gz<br>mESC_DMSO_rep2_H3K4me1_merged_R1.fastq.gz<br>mESC_DMSO_rep1_H3K4me3_merged_R1.fastq.gz<br>mESC_DMSO_rep2_H3K4me3_merged_R1.fastq.gz<br>mESC_DMSO_rep1_H3K79me2_merged_R1.fastq.gz<br>mESC_DMSO_rep2_H3K79me2_merged_R1.fastq.gz<br>mESC_DMSO_rep1_H3K9me3_merged_R1.fastq.gz<br>mESC_DMSO_rep2_H3K9me3_merged_R1.fastq.gz<br>mESC_DMSO_rep1_Input_R1.fastq.gz<br>mESC_DMSO_rep2_Input_R1.fastq.gz<br>mESC_EPZ_rep1_H3K27ac_merged_R1.fastq.gz<br>mESC_EPZ_rep2_H3K27ac_merged_R1.fastq.gz<br>mESC_EPZ_rep1_H3K27me3_merged_R1.fastq.gz<br>mESC_EPZ_rep2_H3K27me3_merged_R1.fastq.gz<br>mESC_EPZ_rep1_H3K36me3_merged_R1.fastq.gz<br>mESC_EPZ_rep2_H3K36me3_merged_R1.fastq.gz<br>mESC_EPZ_rep1_H3K4me1_merged_R1.fastq.gz<br>mESC_EPZ_rep2_H3K4me1_merged_R1.fastq.gz<br>mESC_EPZ_rep1_H3K4me3_merged_R1.fastq.gz<br>mESC_EPZ_rep2_H3K4me3_merged_R1.fastq.gz<br>mESC_EPZ_rep1_H3K79me2_merged_R1.fastq.gz<br>mESC_EPZ_rep2_H3K79me2_merged_R1.fastq.gz<br>mESC_EPZ_rep1_H3K9me3_merged_R1.fastq.gz<br>mESC_EPZ_rep2_H3K9me3_merged_R1.fastq.gz<br>mESC_EPZ_rep1_Input_R1.fastq.gz<br>mESC_EPZ_rep2_Input_R1.fastq.gz<br>NPC48h_DMSO_rep1_H3K27ac_merged_R1.fastq.gz<br>NPC48h_DMSO_rep2_H3K27ac_merged_R1.fastq.gz<br>NPC48h_DMSO_rep1_H3K27me3_merged_R1.fastq.gz<br>NPC48h_DMSO_rep2_H3K27me3_merged_R1.fastq.gz<br>NPC48h_DMSO_rep1_H3K36me3_merged_R1.fastq.gz

NPC48h_DMSO_rep2_H3K36me3_merged_R1.fastq.gz
NPC48h_DMSO_rep1_H3K4me1_merged_R1.fastq.gz
NPC48h_DMSO_rep2_H3K4me1_merged_R1.fastq.gz
NPC48h_DMSO_rep1_H3K4me3_merged_R1.fastq.gz
NPC48h_DMSO_rep2_H3K4me3_merged_R1.fastq.gz
NPC48h_DMSO_rep1_H3K79me2_merged_R1.fastq.gz
NPC48h_DMSO_rep2_H3K79me2_merged_R1.fastq.gz
NPC48h_DMSO_rep1_H3K9me3_merged_R1.fastq.gz
NPC48h_DMSO_rep2_H3K9me3_merged_R1.fastq.gz
NPC48h_DMSO_rep1_Input_R1.fastq.gz
NPC48h_DMSO_rep2_Input_R1.fastq.gz
NPC48h_EPZ_rep1_H3K27ac_merged_R1.fastq.gz
NPC48h_EPZ_rep2_H3K27ac_merged_R1.fastq.gz
NPC48h_EPZ_rep1_H3K27me3_merged_R1.fastq.gz
NPC48h_EPZ_rep2_H3K27me3_merged_R1.fastq.gz
NPC48h_EPZ_rep1_H3K36me3_merged_R1.fastq.gz
NPC48h_EPZ_rep2_H3K36me3_merged_R1.fastq.gz
NPC48h_EPZ_rep1_H3K4me1_merged_R1.fastq.gz
NPC48h_EPZ_rep2_H3K4me1_merged_R1.fastq.gz
NPC48h_EPZ_rep1_H3K4me3_merged_R1.fastq.gz
NPC48h_EPZ_rep2_H3K4me3_merged_R1.fastq.gz
NPC48h_EPZ_rep1_H3K79me2_merged_R1.fastq.gz
NPC48h_EPZ_rep2_H3K79me2_merged_R1.fastq.gz
NPC48h_EPZ_rep1_H3K9me3_merged_R1.fastq.gz
NPC48h_EPZ_rep2_H3K9me3_merged_R1.fastq.gz
NPC48h_EPZ_rep1_Input_R1.fastq.gz
NPC48h_EPZ_rep2_Input_R1.fastq.gz
NPC48h_DMSO_rep1_ATAC_R1.fastq.gz
NPC48h_DMSO_rep2_ATAC_R1.fastq.gz
NPC48h_EPZ_rep1_ATAC_R1.fastq.gz
NPC48h_EPZ_rep2_ATAC_R1.fastq.gz
mESC_DMSO_rep1_RNA-Seq_R1.fastq.gz
mESC_DMSO_rep2_RNA-Seq_R1.fastq.gz
mESC_DMSO_rep3_RNA-Seq_R1.fastq.gz
mESC_EPZ_rep1_RNA-Seq_R1.fastq.gz
mESC_EPZ_rep2_RNA-Seq_R1.fastq.gz
mESC_EPZ_rep3_RNA-Seq_R1.fastq.gz
NPC48h_DMSO_rep1-2_RNA-Seq_R1.fastq.gz
NPC48h_DMSO_rep2-4_RNA-Seq_R1.fasts.gz
NPC48h_DMSO_rep3-5_RNA-Seq_R1.fastq.gz
NPC48h_EPZ_rep1-2a_RNA-Seq_R1.fastq.gz
NPC48h_EPZ_rep2-4a_RNA-Seq_R1.fastq.gz
NPC48h_EPZ_rep3-5a_RNA-Seq_R1.fastq.gz
mESC_DMSO_rep1_H3K27ac_merged_R2.fastq.gz
mESC_DMSO_rep2_H3K27ac_merged_R2.fastq.gz
mESC_DMSO_rep1_H3K27me3_merged_R2.fastq.gz
mESC_DMSO_rep2_H3K27me3_merged_R2.fastq.gz
mESC_DMSO_rep1_H3K36me3_merged_R2.fastq.gz
mESC_DMSO_rep2_H3K36me3_merged_R2.fastq.gz
mESC_DMSO_rep1_H3K4me1_merged_R2.fastq.gz
mESC_DMSO_rep2_H3K4me1_merged_R2.fastq.gz
mESC_DMSO_rep1_H3K4me3_merged_R2.fastq.gz
mESC_DMSO_rep2_H3K4me3_merged_R2.fastq.gz
mESC_DMSO_rep1_H3K79me2_merged_R2.fastq.gz
mESC_DMSO_rep2_H3K79me2_merged_R2.fastq.gz
mESC_DMSO_rep1_H3K9me3_merged_R2.fastq.gz
mESC_DMSO_rep2_H3K9me3_merged_R2.fastq.gz
mESC_DMSO_rep1_Input_R2.fastq.gz
mESC_DMSO_rep2_Input_R2.fastq.gz
mESC_EPZ_rep1_H3K27ac_merged_R2.fastq.gz
mESC_EPZ_rep2_H3K27ac_merged_R2.fastq.gz
mESC_EPZ_rep1_H3K27me3_merged_R2.fastq.gz
mESC_EPZ_rep2_H3K27me3_merged_R2.fastq.gz
mESC_EPZ_rep1_H3K36me3_merged_R2.fastq.gz
mESC_EPZ_rep2_H3K36me3_merged_R2.fastq.gz
mESC_EPZ_rep1_H3K4me1_merged_R2.fastq.gz
mESC_EPZ_rep2_H3K4me1_merged_R2.fastq.gz
mESC_EPZ_rep1_H3K4me3_merged_R2.fastq.gz

mESC_EPZ_rep2_H3K4me3_merged_R2.fastq.gz
mESC_EPZ_rep1_H3K79me2_merged_R2.fastq.gz
mESC_EPZ_rep2_H3K79me2_merged_R2.fastq.gz
mESC_EPZ_rep1_H3K9me3_merged_R2.fastq.gz
mESC_EPZ_rep2_H3K9me3_merged_R2.fastq.gz
mESC_EPZ_rep1_Input_R2.fastq.gz
mESC_EPZ_rep2_Input_R2.fastq.gz
NPC48h_DMSO_rep1_H3K27ac_merged_R2.fastq.gz
NPC48h_DMSO_rep2_H3K27ac_merged_R2.fastq.gz
NPC48h_DMSO_rep1_H3K27me3_merged_R2.fastq.gz
NPC48h_DMSO_rep2_H3K27me3_merged_R2.fastq.gz
NPC48h_DMSO_rep1_H3K36me3_merged_R2.fastq.gz
NPC48h_DMSO_rep2_H3K36me3_merged_R2.fastq.gz
NPC48h_DMSO_rep1_H3K4me1_merged_R2.fastq.gz
NPC48h_DMSO_rep2_H3K4me1_merged_R2.fastq.gz
NPC48h_DMSO_rep1_H3K4me3_merged_R2.fastq.gz
NPC48h_DMSO_rep2_H3K4me3_merged_R2.fastq.gz
NPC48h_DMSO_rep1_H3K79me2_merged_R2.fastq.gz
NPC48h_DMSO_rep2_H3K79me2_merged_R2.fastq.gz
NPC48h_DMSO_rep1_H3K9me3_merged_R2.fastq.gz
NPC48h_DMSO_rep2_H3K9me3_merged_R2.fastq.gz
NPC48h_DMSO_rep1_Input_R2.fastq.gz
NPC48h_DMSO_rep2_Input_R2.fastq.gz
NPC48h_EPZ_rep1_H3K27ac_merged_R2.fastq.gz
NPC48h_EPZ_rep2_H3K27ac_merged_R2.fastq.gz
NPC48h_EPZ_rep1_H3K27me3_merged_R2.fastq.gz
NPC48h_EPZ_rep2_H3K27me3_merged_R2.fastq.gz
NPC48h_EPZ_rep1_H3K36me3_merged_R2.fastq.gz
NPC48h_EPZ_rep2_H3K36me3_merged_R2.fastq.gz
NPC48h_EPZ_rep1_H3K4me1_merged_R2.fastq.gz
NPC48h_EPZ_rep2_H3K4me1_merged_R2.fastq.gz
NPC48h_EPZ_rep1_H3K4me3_merged_R2.fastq.gz
NPC48h_EPZ_rep2_H3K4me3_merged_R2.fastq.gz
NPC48h_EPZ_rep1_H3K79me2_merged_R2.fastq.gz
NPC48h_EPZ_rep2_H3K79me2_merged_R2.fastq.gz
NPC48h_EPZ_rep1_H3K9me3_merged_R2.fastq.gz
NPC48h_EPZ_rep2_H3K9me3_merged_R2.fastq.gz
NPC48h_EPZ_rep1_Input_R2.fastq.gz
NPC48h_EPZ_rep2_Input_R2.fastq.gz
NPC48h_DMSO_rep1_ATAC_R2.fastq.gz
NPC48h_DMSO_rep2_ATAC_R2.fastq.gz
NPC48h_EPZ_rep1_ATAC_R2.fastq.gz
NPC48h_EPZ_rep2_ATAC_R2.fastq.gz
mESC_DMSO_rep1_RNA-Seq_R2.fastq.gz
mESC_DMSO_rep2_RNA-Seq_R2.fastq.gz
mESC_DMSO_rep3_RNA-Seq_R2.fastq.gz
mESC_EPZ_rep1_RNA-Seq_R2.fastq.gz
mESC_EPZ_rep2_RNA-Seq_R2.fastq.gz
mESC_EPZ_rep3_RNA-Seq_R2.fastq.gz
NPC48h_DMSO_rep1-2_RNA-Seq_R2.fastq.gz
NPC48h_DMSO_rep2-4_RNA-Seq_R2.fastq.gz
NPC48h_DMSO_rep3-5_RNA-Seq_R2.fastq.gz
NPC48h_EPZ_rep1-2a_RNA-Seq_R2.fastq.gz
NPC48h_EPZ_rep2-4a_RNA-Seq_R2.fastq.gz
NPC48h_EPZ_rep3-5a_RNA-Seq_R2.fastq.gz
mESC_ATAC_DMSO_rep1_R1.fastq.gz
mESC_ATAC_DMSO_rep1_R2.fastq.gz
mESC_ATAC_DMSO_rep2_R1.fastq.gz
mESC_ATAC_DMSO_rep2_R2.fastq.gz
mESC_ATAC_DMSO_rep3_R1.fastq.gz
mESC_ATAC_DMSO_rep3_R2.fastq.gz
mESC_ATAC_EPZ_rep1_R1.fastq.gz
mESC_ATAC_EPZ_rep1_R2.fastq.gz
mESC_ATAC_EPZ_rep2_R1.fastq.gz
mESC_ATAC_EPZ_rep2_R2.fastq.gz
mESC_ATAC_EPZ_rep3_R1.fastq.gz
mESC_ATAC_EPZ_rep3_R2.fastq.gz
mESC_DMSO_rep1_H3K27ac_merged.filtered.seq_depth_norm.bw

mESC_DMSO_rep2_H3K27ac_merged.filtered.seq_depth_norm.bw
mESC_DMSO_rep1_H3K27me3_merged.filtered.seq_depth_norm.bw
mESC_DMSO_rep2_H3K27me3_merged.filtered.seq_depth_norm.bw
mESC_DMSO_rep1_H3K36me3_merged.filtered.seq_depth_norm.bw
mESC_DMSO_rep2_H3K36me3_merged.filtered.seq_depth_norm.bw
mESC_DMSO_rep1_H3K4me1_merged.filtered.seq_depth_norm.bw
mESC_DMSO_rep2_H3K4me1_merged.filtered.seq_depth_norm.bw
mESC_DMSO_rep1_H3K4me3_merged.filtered.seq_depth_norm.bw
mESC_DMSO_rep2_H3K4me3_merged.filtered.seq_depth_norm.bw
mESC_DMSO_rep1_H3K79me2_merged.filtered.seq_depth_norm.bw
mESC_DMSO_rep2_H3K79me2_merged.filtered.seq_depth_norm.bw
mESC_DMSO_rep1_H3K9me3_merged.filtered.seq_depth_norm.bw
mESC_DMSO_rep2_H3K9me3_merged.filtered.seq_depth_norm.bw
mESC_DMSO_rep1_Input.filtered.seq_depth_norm.bw
mESC_DMSO_rep2_Input.filtered.seq_depth_norm.bw
mESC_EPZ_rep1_H3K27ac_merged.filtered.seq_depth_norm.bw
mESC_EPZ_rep2_H3K27ac_merged.filtered.seq_depth_norm.bw
mESC_EPZ_rep1_H3K27me3_merged.filtered.seq_depth_norm.bw
mESC_EPZ_rep2_H3K27me3_merged.filtered.seq_depth_norm.bw
mESC_EPZ_rep1_H3K36me3_merged.filtered.seq_depth_norm.bw
mESC_EPZ_rep2_H3K36me3_merged.filtered.seq_depth_norm.bw
mESC_EPZ_rep1_H3K4me1_merged.filtered.seq_depth_norm.bw
mESC_EPZ_rep2_H3K4me1_merged.filtered.seq_depth_norm.bw
mESC_EPZ_rep1_H3K4me3_merged.filtered.seq_depth_norm.bw
mESC_EPZ_rep2_H3K4me3_merged.filtered.seq_depth_norm.bw
mESC_EPZ_rep1_H3K79me2_merged.filtered.seq_depth_norm.bw
mESC_EPZ_rep2_H3K79me2_merged.filtered.seq_depth_norm.bw
mESC_EPZ_rep1_H3K9me3_merged.filtered.seq_depth_norm.bw
mESC_EPZ_rep2_H3K9me3_merged.filtered.seq_depth_norm.bw
mESC_EPZ_rep1_Input.filtered.seq_depth_norm.bw
mESC_EPZ_rep2_Input.filtered.seq_depth_norm.bw
NPC48h_DMSO_rep1_H3K27ac_merged.filtered.seq_depth_norm.bw
NPC48h_DMSO_rep2_H3K27ac_merged.filtered.seq_depth_norm.bw
NPC48h_DMSO_rep1_H3K27me3_merged.filtered.seq_depth_norm.bw
NPC48h_DMSO_rep2_H3K27me3_merged.filtered.seq_depth_norm.bw
NPC48h_DMSO_rep1_H3K36me3_merged.filtered.seq_depth_norm.bw
NPC48h_DMSO_rep2_H3K36me3_merged.filtered.seq_depth_norm.bw
NPC48h_DMSO_rep1_H3K4me1_merged.filtered.seq_depth_norm.bw
NPC48h_DMSO_rep2_H3K4me1_merged.filtered.seq_depth_norm.bw
NPC48h_DMSO_rep1_H3K4me3_merged.filtered.seq_depth_norm.bw
NPC48h_DMSO_rep2_H3K4me3_merged.filtered.seq_depth_norm.bw
NPC48h_DMSO_rep1_H3K79me2_merged.filtered.seq_depth_norm.bw
NPC48h_DMSO_rep2_H3K79me2_merged.filtered.seq_depth_norm.bw
NPC48h_DMSO_rep1_H3K9me3_merged.filtered.seq_depth_norm.bw
NPC48h_DMSO_rep2_H3K9me3_merged.filtered.seq_depth_norm.bw
NPC48h_DMSO_rep1_Input.filtered.seq_depth_norm.bw
NPC48h_DMSO_rep2_Input.filtered.seq_depth_norm.bw
NPC48h_EPZ_rep1_H3K27ac_merged.filtered.seq_depth_norm.bw
NPC48h_EPZ_rep2_H3K27ac_merged.filtered.seq_depth_norm.bw
NPC48h_EPZ_rep1_H3K27me3_merged.filtered.seq_depth_norm.bw
NPC48h_EPZ_rep2_H3K27me3_merged.filtered.seq_depth_norm.bw
NPC48h_EPZ_rep1_H3K36me3_merged.filtered.seq_depth_norm.bw
NPC48h_EPZ_rep2_H3K36me3_merged.filtered.seq_depth_norm.bw
NPC48h_EPZ_rep1_H3K4me1_merged.filtered.seq_depth_norm.bw
NPC48h_EPZ_rep2_H3K4me1_merged.filtered.seq_depth_norm.bw
NPC48h_EPZ_rep1_H3K4me3_merged.filtered.seq_depth_norm.bw
NPC48h_EPZ_rep2_H3K4me3_merged.filtered.seq_depth_norm.bw
NPC48h_EPZ_rep1_H3K79me2_merged.filtered.seq_depth_norm.bw
NPC48h_EPZ_rep2_H3K79me2_merged.filtered.seq_depth_norm.bw
NPC48h_EPZ_rep1_H3K9me3_merged.filtered.seq_depth_norm.bw
NPC48h_EPZ_rep2_H3K9me3_merged.filtered.seq_depth_norm.bw
NPC48h_EPZ_rep1_Input.filtered.seq_depth_norm.bw
NPC48h_EPZ_rep2_Input.filtered.seq_depth_norm.bw
NPC48h_DMSO_rep1_ATAC.filtered.PeakScaled.bw
NPC48h_DMSO_rep2_ATAC.filtered.PeakScaled.bw
NPC48h_EPZ_rep1_ATAC.filtered.PeakScaled.bw
NPC48h_EPZ_rep2_ATAC.filtered.PeakScaled.bw
mESC_DMSO_rep1_RNA-Seq.RPKM.bw

mESC_DMSO_rep2_RNA-Seq.RPKM.bw
mESC_DMSO_rep3_RNA-Seq.RPKM.bw
mESC_EPZ_rep1_RNA-Seq.RPKM.bw
mESC_EPZ_rep2_RNA-Seq.RPKM.bw
mESC_EPZ_rep3_RNA-Seq.RPKM.bw
NPC48h_DMSO_rep1-2_RNA-Seq.RPKM.bw
NPC48h_DMSO_rep2-4_RNA-Seq.RPKM.bw
NPC48h_DMSO_rep3-5_RNA-Seq.RPKM.bw
NPC48h_EPZ_rep1-2a_RNA-Seq.RPKM.bw
NPC48h_EPZ_rep2-4a_RNA-Seq.RPKM.bw
NPC48h_EPZ_rep3-5a_RNA-Seq.RPKM.bw
mESC_ATAC_DMSO_rep1.filtered.PeakScaled.bw
mESC_ATAC_DMSO_rep2.filtered.PeakScaled.bw
mESC_ATAC_DMSO_rep3.filtered.PeakScaled.bw
mESC_ATAC_EPZ_rep1.filtered.PeakScaled.bw
mESC_ATAC_EPZ_rep2.filtered.PeakScaled.bw
mESC_ATAC_EPZ_rep3.filtered.PeakScaled.bw

Genome browser session (e.g. UCSC)

No longer applicable.

Methodology

Replicates

ChIP-seq: 2 replicates per condition and per cell type;
RNA-seq: 3 replicates per condition and per cell type;
ATAC-seq: 2 (NPC48h) / 3 (mESC) replicates per condition;

Sequencing depth

A detailed report providing sequencig depth for each experiment is available at https://github.com/FrancescoFerrari88/code_DOT1L_paper/tree/master/multiQC_ConfigParameters

Antibodies

H3K27ac: Diagenode, C1541096, lot A1723-041D, concentration: 2.84 µg/µL, used: 1ul per 100,000 cells
H3K27me3: Diagenode, C15410195, lot A1811-001P, concentration: 1.91 µg/µL, used: 1ul per 100,000 cells
H3K36me3: Diagenode, C15410192, lot A1847-001P, concentration: 1.19 µg/µL, used: 1ul per 100,000 cells
H3K4me1: Diagenode, C15410194, lot A1863-001D, concentration: 0.91 µg/µL, used: 1ul per 100,000 cells
H3K4me3: Diagenode, C15410003, lot A5051-001P, concentration: 1 µg/µL, used: 1ul per 100,000 cells
H3K79me2: Abcam, ab3594, concentration: 2 µg/µL, used: 1ul per 100,000 cells
H3K9me3: Diagenode, C15410193, lot A1671-001P, concentration: 2.69 µg/µL, used: 1ul per 100,000 cells

Peak calling parameters

Peak calling was performed with MACS2 (v. 2.1.2), q-value = 0.001 (default parameter in snakePipes v. 1.1.1)

Data quality

The overall quality of the generated ChIP-seq data were initially assessed using deeptools' plotFingerprint. FRiP score was calculated for each experiment. Called peaks were visually inspected in IGV to assess if predicted peaks co-localized with local enrichment of each mark.

Software

Custom code used to analyze the data is available at https://github.com/FrancescoFerrari88/code_DOT1L_paper
